# Antler stem cell-derived exosomes restore periodontal homeostasis in a rat model with diabetic periodontitis through enhancing ROS scavenging and osteogenesis

**DOI:** 10.1038/s41420-025-02800-6

**Published:** 2025-11-03

**Authors:** Qianqian Guo, Sicong Ren, Antonio Libonati, Jiping Li, Zhen Wang, Jing Ren, Guokun Zhang, Linlin Gao, Hengxing Ba, Yuqin Shen, Chunyi Li

**Affiliations:** 1 Institute of Antler Science and Product Technology, Changchun Sci-Tech University, Changchun, Jilin China; 2 School of Stomatology, Jilin University, Changchun, Jilin China; 3 Department of Clinical Sciences and Translational Medicine, University of Rome Tor Vergata, Rome, Italy; 4 College of Life Science, Jilin Agricultural University, Changchun, Jilin China; 5 Department of Periodontology, Affiliated Stomatology Hospital of Guangzhou Medical University, Guangzhou, Guangdong China

**Keywords:** Regeneration, Regenerative medicine

## Abstract

Diabetes mellitus (DM) exacerbates periodontitis as the high-glucose (HG) environment aggravates local inflammation and periodontal bone resorption. Restoring periodontal homeostasis and promoting periodontal bone repair/regeneration are major challenges for the treatment of diabetic periodontitis. This study introduces antler stem cell-derived exosomes (AnSC-exos) as a potent therapeutic for treating diabetic periodontitis via leveraging the shared cranial neural crest cell (CNCC) origin of antlers and periodontal tissues. Using a rat model of diabetic periodontitis, we demonstrate that AnSC-exos effectively alleviate tissue abnormalities and alveolar bone destruction and resorption in periodontitis under DM conditions; the outcome was significantly more potent than human bone marrow mesenchymal stem cell exosomes (hBMSC-exos). Mechanistically, AnSC-exos exhibited dual regenerative actions: (1) restoring the osteogenic ability of resident MSCs by not only reversing high glucose (HG)-induced suppression of proliferation and migration, but more importantly, enhancing cell survival, reducing cell death, and strengthening differentiation toward osteogenic lineages under HG conditions; and (2) attenuating inflammation through potently scavenging excessive ROS production induced by HG, and inhibiting HG-mediated p65 nuclear translocation, thereby leading to a reduced M1/M2 macrophage ratio. In conclusion, the superior efficacy of AnSC-exos highlights their tissue-specific regenerative advantage and establishes AnSC-exos as a promising cell-free therapy that simultaneously targets osteogenic impairment and ROS-driven inflammation in diabetic periodontitis. Further characterization of active components within the exosomes holds significant promise for developing effective clinical treatments for diabetic periodontitis.

## Introduction

Periodontitis is a highly prevalent inflammatory disease, ranking third among noncommunicable illnesses, behind cancer and cardiovascular disease [1]. Periodontitis is characterized by the formation of periodontal pockets and the resorption of alveolar bone, ultimately leading to tooth loss. Moreover, periodontitis is the sixth major complication of diabetes mellitus (DM). Once patients suffer from DM, the destruction of periodontal bone is further accelerated [2, 3], resulting in severe periodontitis. It is estimated that 10.8% of the world’s population suffers from severe periodontitis [4]. Periodontitis under diabetic conditions shows less favorable responses to conventional treatment modalities than non-diabetic periodontitis. Mechanistically, aberrant glucose metabolism causes the overproduction of reactive oxygen species (ROS) [5, 6], leading to an imbalance in periodontal homeostasis, which in turn inhibits periodontal bone formation and regeneration [7, 8]. Therefore, developing strategies reducing ROS overproduction and enhancing osteogenesis is critical for the repair/regeneration of diabetic periodontal tissue.

Accumulated studies have revealed that mesenchymal stem cells (MSCs) have significant efficacies for treating a variety of human diseases, including periodontitis and DM [9–12]. Locally injected MSCs can effectively repair periodontal defects in a rat model of periodontitis [13]. Growing evidence supports that MSCs exert antioxidant properties in a variety of animal disease models, which may be achieved by via scavenging ROS [14, 15]. These findings demonstrate the potential application of MSCs in treating periodontitis in DM patients. Among various MSCs used for periodontal diseases, dental mesenchymal cells (DMCs), including periodontal ligament stem cells and dental pulp stem cells, have proven to be the most effective ones for this purpose in vivo [16]. However, the limited sources, the inconsistency of donor quality, and the painful process of collection have hindered their application in the field.

Deer antlers are the only mammalian appendage that, once lost, can fully regenerate [16–19]. It is known that antler regeneration is a stem cell-based process, and antler cells originate from cranial neural crest-derived ectomesenchymal cells during embryo development [20–22], as do the teeth [16, 21–23]. Our recent studies have shown that there are shared gene expression profiles between AnSCs and deer DMCs, indicating a common developmental origin [24]. AnSCs and DMCs are both neural crest-derived cells, and AnSCs have the power to regenerate a whole organ. So it may be possible to harness the proliferation and differentiation potential of AnSCs to induce regeneration of damaged periodontal tissue in DM. Recently, we applied AnSC-conditioned media (AnSC-CM) to treat periodontitis in an animal model and found that AnSC-CM significantly reduced alveolar bone resorption and inhibited inflammation in the gingival tissue [16, 25, 26], and the outcome of AnSC-CM treatment was significantly better than human bone marrow mesenchymal stem cell-CM (hBMSC-CM), suggesting that paracrine factors of AnSCs have significant therapeutic efficacy on periodontitis. However, the putative paracrine factors have not been identified.

Exosomes, a type of small extracellular vesicle with diameters of 30–150 nm, are secreted by cells [27–29]. The main functions of exosomes are information transmission, antigen presentation, regulation of immune and target cells, and tissue regeneration [28]. Recently, exosomes have been recognized as the main mediators in the therapeutic function of MSCs [30, 31]. Latest studies have confirmed that MSC-derived exosomes can promote alveolar bone defect repair and periodontal tissue regeneration in rats [32]. Herein, we speculate that AnSC-derived exosomes (AnSC-exos) would be responsible for the excellent therapeutic efficacy of AnSC-CM on periodontitis in animal models. The aims of the present study were two-fold: (1) to examine the efficacy of AnSC-exos on severe periodontitis of DM rats in comparison with hBMSC-exos; and (2) to explore the molecular mechanisms underlying the effective treatment of AnSC-exos on diabetic periodontitis through an in vitro approach. We found that AnSC-exos effectively restored periodontal homeostasis by attenuating excessive periodontal inflammation and rescued alveolar bone destruction and resorption in periodontitis under DM conditions. We also demonstrated that AnSC-exos played a role in inhibiting the ROS/NF-κB pathway. In conclusion, our work has discovered a promising cell-free remedy for periodontal tissue repair/regeneration in DM and opens up a new avenue for the development of new drugs based on AnSC-exos in the clinical setting.

## Results

### AnSC-exos rescues alveolar bone loss in periodontitis of diabetic rats

**Fig. 1:**
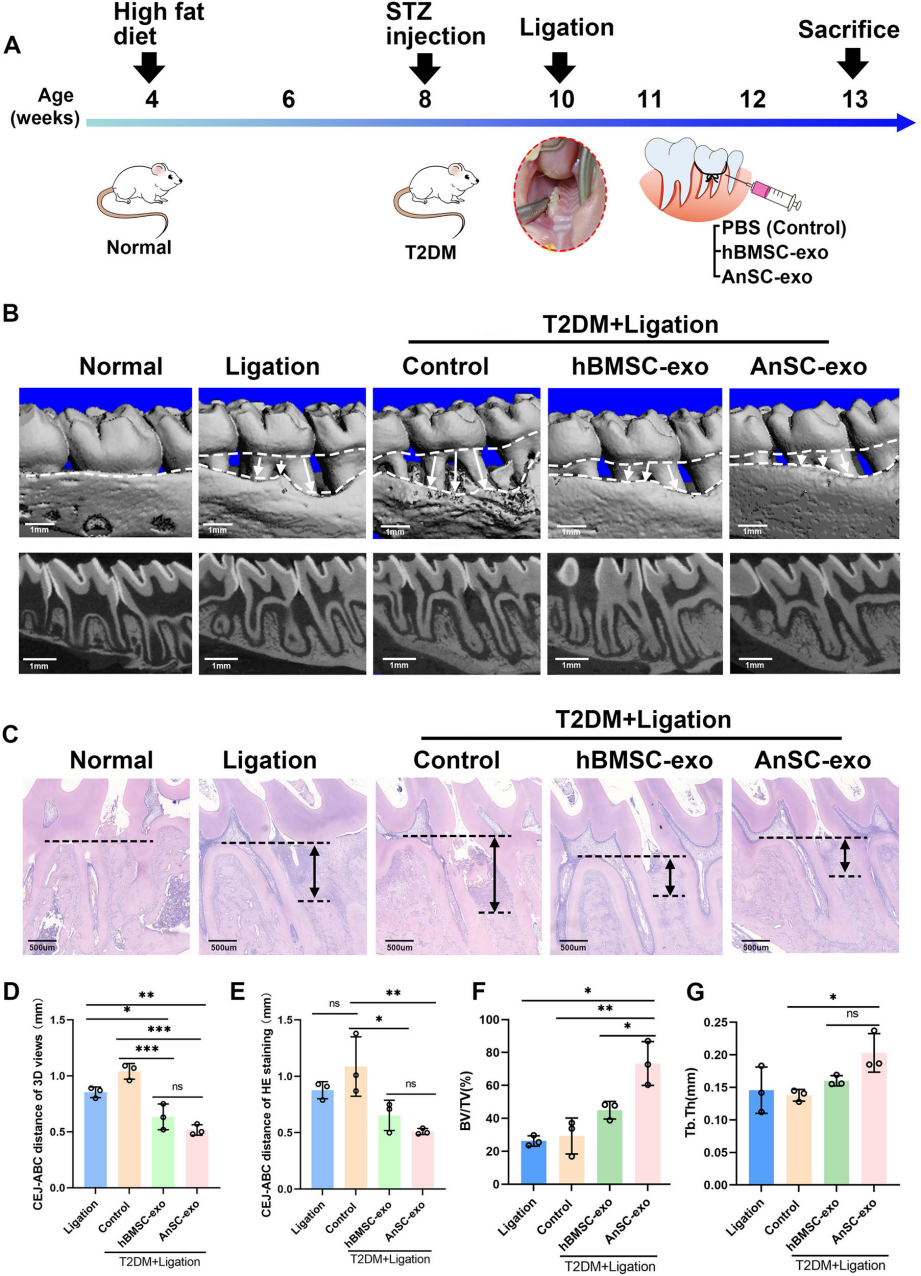
Effects of AnSC-exos on diabetic periodontitis in vivo in rats. **A** Schematic diagram illustrating the time frame of the in vivo study. **B** Micro-CT images of maxillary alveolar bone. The upper image in each group displays the 3D reconstruction image, and the lower images represent the bucco-palatal sagittal slices of Micro-CT. Note that the Control group exhibited the most pronounced bone loss, while after the treatment of AnSC-exos and hBMSC-exos, bone loss was significantly reduced. **C** HE staining images of periodontal tissue. The two-way arrow refers to the distance between the alveolar bone crest and the cementoenamel junction (CEJ-ABC). **D**, **E** Quantitative analysis of the CEJ-ABC distance in Micro-CT and HE staining. Comparisons of parameters of the periodontal bone tissue in different groups: bone volume/tissue volume (**F**, BV/TV) and average thickness of bone trabecula (**G**, Tb.Th). Error bar represents the mean ± SD (*n* = 3); * *p* < 0.05; ** *p* < 0.01, *** *p* < 0.001.

As illustrated in schematic Fig. 1, the in vivo therapeutic efficacy of AnSC-exos was evaluated in diabetic rats with ligature-induced periodontitis. Two weeks after Streptozotocin (STZ) injection, a stable blood glucose level above 11.1 mmol/L was reached in all rats. Following the ligation of the second maxillary molar, alveolar bone destruction and resorption were observed macroscopically. Therefore, the periodontitis model in type 2 DM rats (T2DM) was successfully established (Fig. 1A). The 3D reconstruction based on the micro-computed tomography (Micro-CT) scan results (Fig. 1B) clearly showed alveolar bone loss after ligation in both normal and diabetic rats. The diabetic periodontitis group (Control) exhibited the most pronounced bone loss, while after the treatments with AnSC-exos or hBMSC-exos, the degree of bone loss was significantly decreased. Analysis of the distance between the alveolar bone crest and the cementoenamel junction (CEJ-ABC), the indicator of alveolar bone destruction, showed that the distance was the widest in the Control group, significantly narrower in the AnSC-exos and hBMSC-exos groups than the Control group (*P* < 0.05), and the narrowest in the AnSC-exos group (Fig. 1D). In addition, possessed bone volume/tissue volume (BV/TV) in the AnSC-exos group was significantly higher than the rest of the groups; notably, this value in the AnSC-exos group was significantly higher than that in the hBMSC-exos (Fig. 1F). The average thickness of bone trabeculae (TbTh) in the AnSC-exos group was also significantly greater than the Control group, while the difference between the hBMSC-exos group and the rest of the groups did not reach statistical significance (Fig. 1G). The microCT results suggest a positive effect of AnSC-exos treatment on alleviating bone loss in periodontitis of diabetic rats, and the therapeutic effect is even better than that of hBMSC-exos in some key aspects.

Consistent with the results of micro-CT, histological examination (Fig. 1C) revealed the Control group had the longest CEJ-ABC distance, while both AnSC-exos and hBMSC-exos had significant shorter CEJ-ABC distance compared to the Control group. Analysis of the CEJ-ABC distance showed that the mean value was the smallest in the AnSC-exos group, and significantly shorter than those of both the Ligation and Control groups, but the difference with the hBMSC-exos group did not reach statistical significance (Fig. 1E). As described in Fig. 1C, infiltration of extensive inflammatory cells was observed in both the Ligation and Control groups, especially in the Control group, whereas the number of inflammatory cells was significantly less in both the AnSC-exos and hBMSC-exos groups. In summary, our in vivo experiments demonstrate that AnSC-exos treatment can effectively reduce tissue abnormalities and reduce alveolar bone destruction and resorption in periodontitis under the DM condition.

### AnSC-exos attenuates the level of inflammation in the induced-periodontitis in diabetic rats

**Fig. 2:**
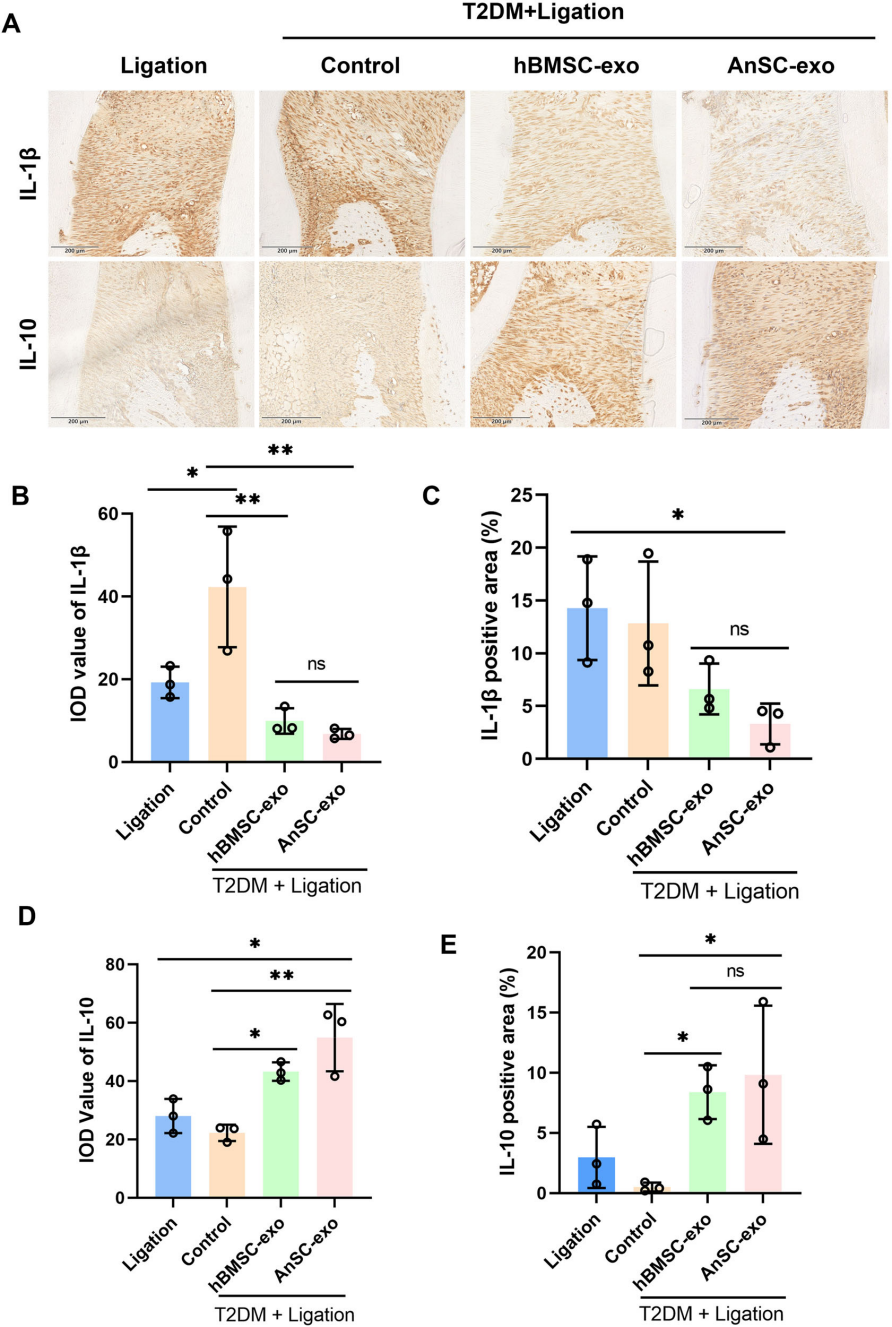
Effects of AnSC-exos on periodontal inflammation in diabetic rats with experimental periodontitis. **A** The immunohistochemical staining of IL-1β and IL-10 in the gingival tissues. **B**, **C** Quantitative analysis of the integratedoption density (IOD) value and positive area occupancy (area %) of IL-1β, based on the results of (**A**). Note that the expression levels of IL-1β in AnSC-exos group was lower, but that of IL-10 was higher than that in the Ligation and Control groups. **D**, **E** Quantitative analysis of the IOD value and positive area of IL-10, based on the results of (**A**). Error bar represents the mean ± SD (*n* = 3). * *p* < 0.05; ** *p* < 0.01.

As reported, the long-term inflammatory environment in the state of diabetes causes local inflammation [7, 33, 34]. Therefore, controlling inflammation is one of the key factors in inhibiting periodontal bone resorption and enhancing bone regeneration. As shown in Fig. 2A, strong expression of IL-1β was detected in both Ligation and Control groups, while only a negligible expression level of IL-1β was detected in both AnSC-exos and hBMSC-exos groups, indicating that both AnSC-exos and hBMSC-exos treatments effectively reversed the inflammatory state. In contrast, both the AnSC-exos and hBMSC-exos groups exhibited significantly higher expression levels of IL-10 (anti-inflammatory cytokine) compared to those in both the Ligation and Control groups. Quantitative analysis showed that the expression level of IL-1β in the AnSC-exos group was significantly lower, while that of IL-10 was significantly higher than those in both the Ligation and Control groups; there was no significant difference between the AnSC-exos and hBMSC-exos groups (Fig. 2B–E). Therefore, we conclude that MSCs-derived exosomes, including AnSC-exos and hBMSC-exos, can effectively restore periodontal homeostasis, highly likely via attenuating excessive periodontal inflammation under the DM condition.

### AnSC-exos promotes rBMSC cell proliferation and migration under high glucose condition in vitro

**Fig. 3:**
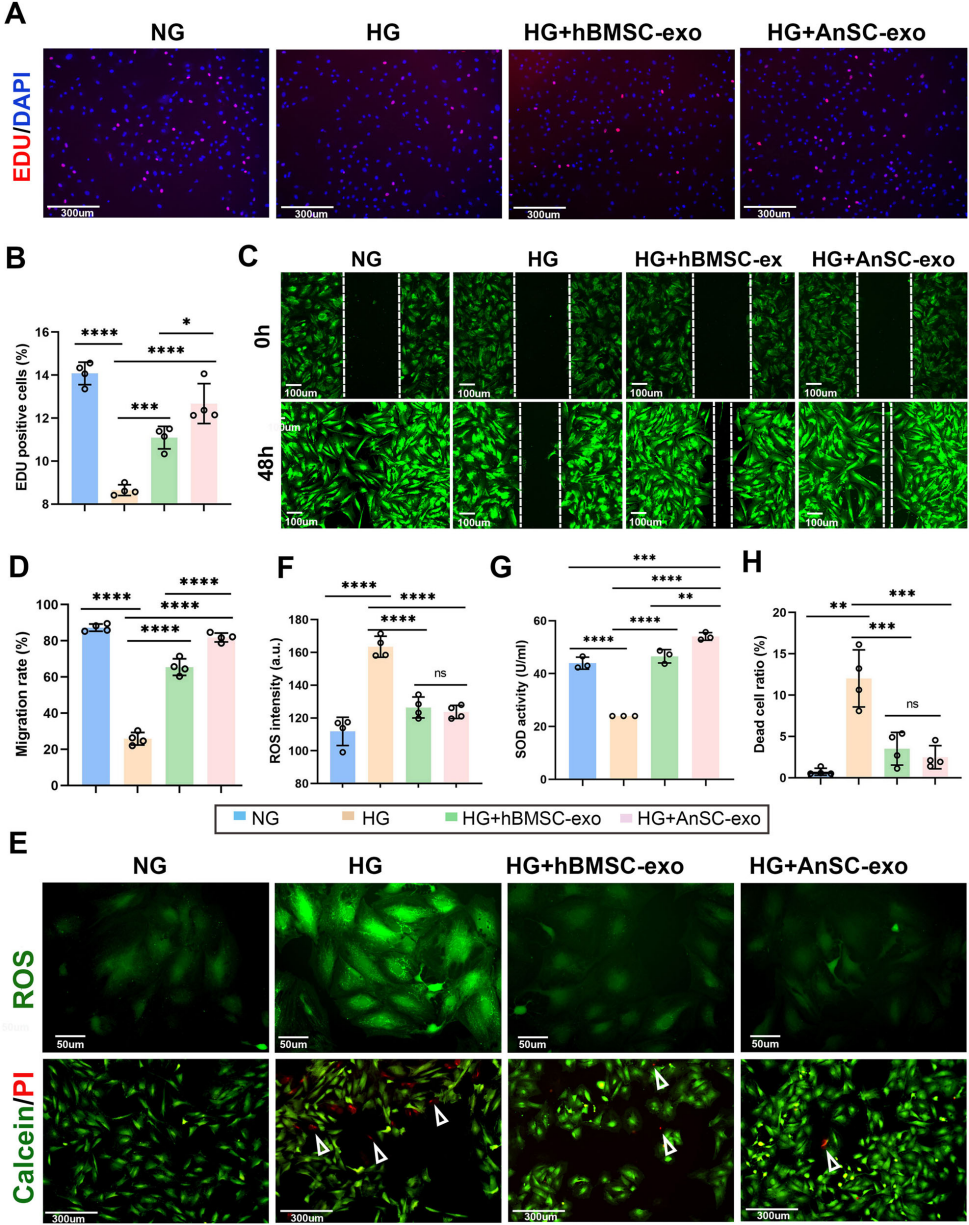
Effects of AnSC-exos on cell survival, cell migration and ROS production of rBMSC under the high glucose (HG) condition. **A** Mitogenic effect of AnSC-exos on rBMSCs via EDU incorporation assays. Red fluorescence: EDU-positive cells. **B** Percentage of EDU-positive cells. Note that the AnSC-exos group had a higher percentage of EDU-positive cells than hBMSC-exos. Error bar represents the mean ± SD (*n* = 4). * *p* < 0.05; ***p < 0.001; ****p < 0.0001. **C** Representative images of scratch assays of rBMSCs in the different groups. Note that in the AnSC-exos group, the scratched spaces had almost filled via migration of rBMSCs 48 h after scratch. **D** Quantification of migration rates of rBMSCs in the different groups, based on the results of (**C**). Error bar represents the mean ± SD (*n* = 4). ****p < 0.0001. **E** ROS and live/dead staining of rBMSCs. The upper panel displays the representative fluorescent images of the intracellular ROS in rBMSCs. The lower panel displays the representative images of live/dead staining of rBMSCs. Red fluorescence bodies represent dead cells, and green fluorescence bodies represent living cells. **F** Quantitative analysis of ROS intensity, based on the results of (**E**). Error bar represents the mean ± SD (*n* = 4). ****p < 0.0001. **G** SOD activity in the different groups. Error bar represents the mean ± SD (*n* = 3). **p < 0.01; ***p < 0.001; ****p < 0.0001. **H** Dead cell ratio of rBMSCs under the HG condition in the different groups. Error bar represents the mean ± SD (*n* = 4). **p < 0.01; ***p < 0.001.

It is known that recruitment of endogenous bone marrow mesenchymal stem cells (BMSCs) to the defect area is the first critical step for in situ bone regeneration [2, 35, 36]. However, we know that cell proliferation and migration are negatively impacted in diabetes [2, 37, 38]. Herein, we sought to assess the effects of AnSC-exos on cell proliferation and migration of rat BMSCs (rBMSCs) under the high glucose (HG) condition. As shown in Fig. 3A, the number of EDU-positive cells (proliferating cells) in the HG group was significantly decreased compared to that in the normal glucose (NG) group. After the treatment of AnSC-exos or hBMSC-exos, the number of proliferating cells was significantly increased in the HG condition. Quantitative analysis showed that the number of proliferating cells in the AnSC-exos group was significantly higher than that in the HG group, as well as the HG+hBMSC-exos group, and was even comparable to that of the NG group (Fig. 3B).

In the migration assay, the lowest migration rate of the rBMSCs was found in the HG group among all other groups (Fig. 3C, D). The migration rates of the rBMSCs in both HG+AnSC-exos and HG+hBMSC-exos groups were significantly higher compared to that in the HG group; notably, the migration rate of rBMSCs in the HG+AnSC-exos group was significantly higher than that in the HG+hBMSC-exos group (Fig. 3D), and the gap in the HG+AnSC-exos group was essentially filled 48 h after the scratch. Overall, our results reveal that AnSC-exos can reverse the inhibition of cell proliferation and migration in the HG condition, and thus hold great potential in MSC recruitment for bone regeneration in diabetic periodontitis.

### AnSC-exos inhibits overproduction of ROS and ROS-induced cell death in rBMSCs in the HG condition in vitro

It is known that in T2DM, aberrant glucose metabolism causes ROS overproduction [39], so here we sought to determine the ROS scavenging activities of AnSC-exos using the intracellular ROS fluorescent probe DCFH-DA. As shown in Fig. 3E, a distinctive pattern of green fluorescence was observed in the HG group, indicating the presence of a high level of intracellular ROS; in contrast, there was a negligible green signal detected in the NG group. In both HG+AnSC-exos and HG+hBMSC-exos groups, the ROS signal was significantly lower compared to that in the HG group. Quantitative analysis results showed that the AnSC-exos group was the lowest in ROS level under the HG condition, and significantly lower than that in the HG group, but there was no significant difference with the HG+hBMSC-exos group (Fig. 3F). Given that superoxide dismutase (SOD) controls the level of ROS and eliminates the potential toxicity of ROS [40], we next examined the effects of AnSC-exos on the expression level of SOD in the HG condition. As shown in Fig. 3G, the SOD level was the lowest in the HG group among the rest of the groups. Treatment of AnSC-exos or hBMSC-exos significantly increased the SOD levels under the HG condition; notably, the SOD level in the AnSC-exos group was significantly higher than that in the hBMSC-exos group.

Under normal conditions, ROS plays crucial roles in diverse cellular functions, but excessive ROS accumulation can lead to oxidative stress, potentially causing cell death [41]. To investigate whether AnSC-exos could alleviate cell death via eliminating excessive ROS, we performed live/dead staining. As shown in Fig. 3H, red fluorescence was observed in the HG group, indicating the existence of dead cells; in contrast, there were almost no detectable dead cells in the rest of the groups. Quantitative analysis results showed that the dead-to-live cell ratio was the highest in the HG group, but was significantly lower in both AnSC-exos and hBMSC-exos groups. Overall, our results reveal that AnSC-exos can effectively remove excessive ROS in the HG condition, at least partially through increasing the SOD expression level, and thus reversing ROS-induced cell death.

### AnSC-exos exhibits osteogenic effects on rBMSCs under the HG condition in vitro

**Fig. 4:**
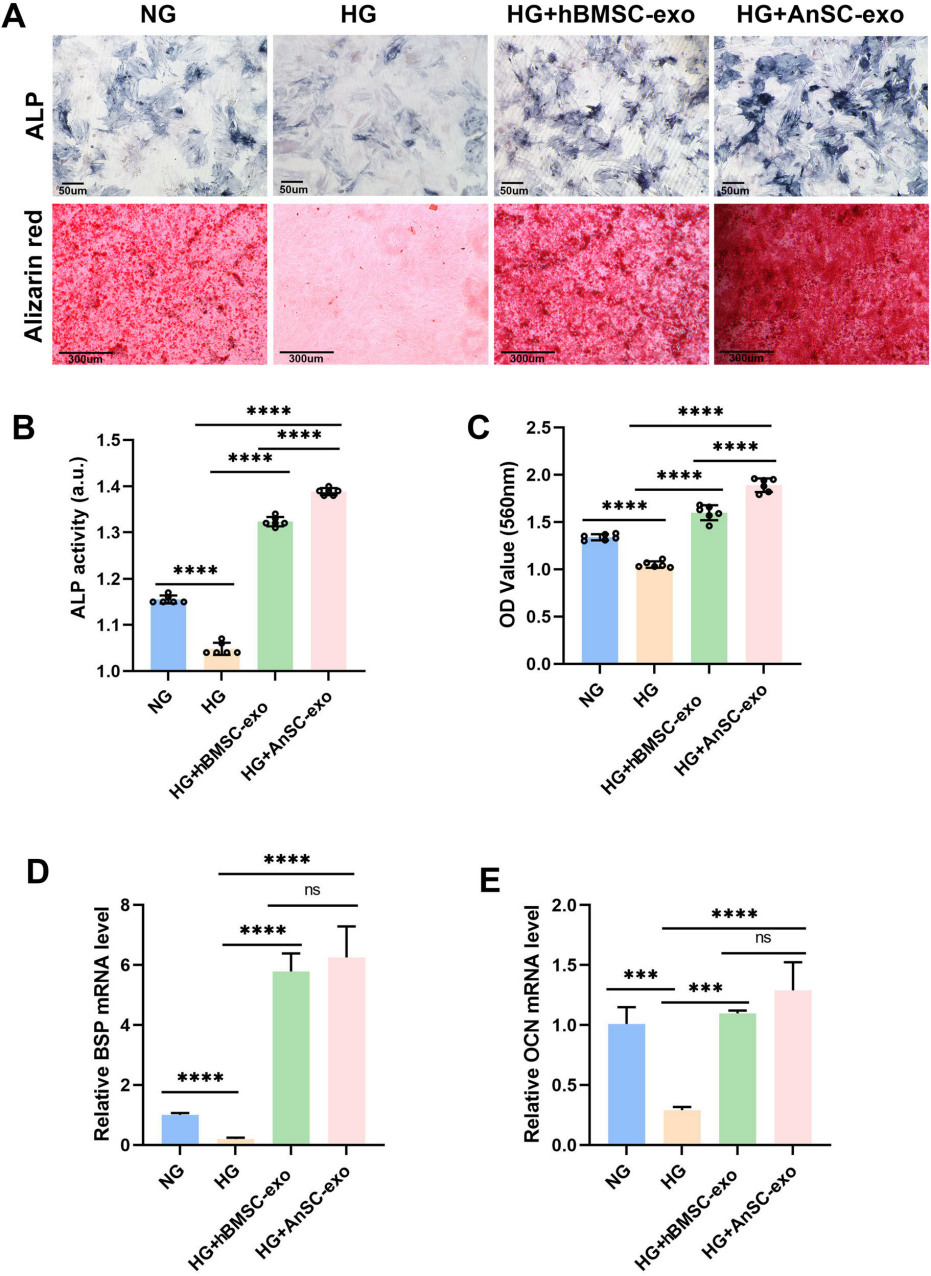
Effects of AnSC-exos on osteogenic differentiation of rBMSCs. **A** Representative images of ALP staining (upper) and mineralized nodules (red color) formed by rBMSCs (lower) in the osteogenic induction media under the HG condition. **B** Analysis of ALP activity in rBMSCs 7 days after osteogenic induction culture. Error bar represents the mean ± SD (*n* = 6). ****p < 0.0001. **C** Mineralization quantification based on the results of (**C**). Error bar represents the mean ± SD (*n* = 6). ****p < 0.0001. **D**, **E** Relative expression levels of osteogenic genes on day 7 in the osteogenic induction media, including BSP and OCN. Error bar represents the mean ± SD (*n* = 3). ***, *p* < 0.001; ****p < 0.0001.

It is known that MSCs exhibit reduced osteogenic differentiation capability under diabetic conditions [42]. Herein, we sought to explore whether AnSC-exos could alleviate this impaired osteogenic differentiation. In the osteogenic induction assay, we found that lighter staining of ALP was detected in the HG group than in the rest of the groups (Fig. 4A). The HG+AnSC-exos group displayed deeper staining of ALP than the rest of the groups. ALP activity assay results showed the same trends (Fig. 4B), i.e., ALP level was the highest in the HG+AnSC-exos group and the lowest in the HG group; notably, the ALP level in the AnSC-exos group was significantly higher than that in the hBMSC-exos group. Alizarin red staining results showed that rBMSCs in the HG+AnSC-exos group formed more and larger mineralized nodules than the rest of the groups; notably, the number of nodules in the HG+AnSC-exos group was significantly greater than that in the HG+hBMSC-exos group (Fig. 4A, C). Furthermore, expression levels of Bone Sialoprotein (BSP) and Osteocalcin (OCN) in rBMSCs of the HG group were significantly lower than those of the NG group. Treatment with AnSC-exos or hBMSC-exos under the HG condition significantly increased expression levels of these genes, and the levels reached in these groups even exceeded those in the NG group (Fig. 4D, E). All these results indicate that AnSC-exos can effectively reverse the negative effects of HG on osteogenesis and exhibit strong potential of driving rBMSCs to differentiate toward osteogenic lineage cells, and this potential was even stronger than that of hBMSC-exos.

### AnSC-exos plays anti-inflammatory role via mediating ROS/NFκB signaling pathways under the HG condition

**Fig. 5:**
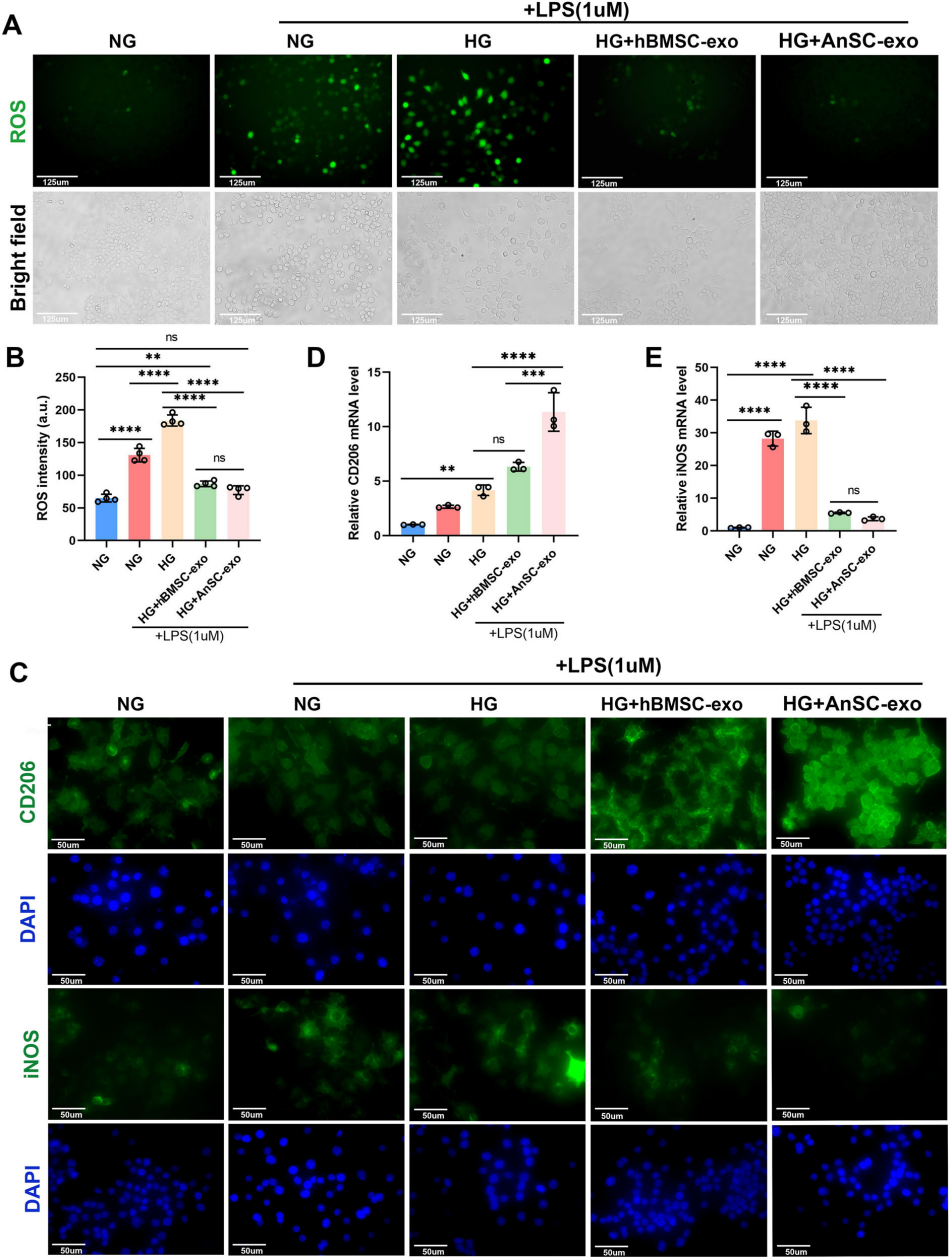
Effects of AnSC-exos on ROS production and polarization of RAW264.7 cells under the HG condition. **A** ROS staining of RAW264.7 cells. The representative fluorescence images of intracellular ROS are shown in the upper figure, and the corresponding images under bright field are shown in the lower figure. **B** Quantitative analysis of ROS intensity, based on the results of (**A**). Error bar represents the mean ± SD (*n* = 4). **p < 0.01; ****p < 0.0001. **C** Immunofluorescent staining of CD206 (green) and iNOS (green) protein in RAW264.7 cells in the different treatment groups. **D**, **E** Relative expression levels of CD206 and iNOS in RAW264.7 cells in the different treatment groups. Error bar represents the mean ± SD (*n* = 4). **, *p* < 0.01;***p < 0.001; ****p < 0.0001.

Excessive ROS production in the diabetic condition causes cells to be in a long-term inflammatory state, which has negative impacts on the balance of ratio of M1 and M2 macrophages [34, 43]. In this study, we evaluated the effects of AnSC-exos on macrophage polarization under the HG condition using the cell line RAW264.7 in vitro. The RAW264.7 cells were cultured in the HG medium supplemented with lipopolysaccharide (LPS) to imitate the inflammatory microenvironment of diabetes. As shown in Fig. 5A, B, ROS overproduction in RAW264.7 cells was induced under the HG condition supplemented with LPS compared to the NG group. In contrast, treatment with AnSC-exos or hBMSC-exos significantly reduced the ROS levels caused by HG + LPS, and the results were even comparable to those of the NG group. Immunofluorescent staining results (Fig. 5C) revealed that the expression level of CD206 (M2 macrophage marker) in the AnSC-exos-treated group was significantly higher, while the expression level of iNOS (M1 macrophage marker) was significantly lower than that in the rest of the groups, suggesting that AnSC-exos can effectively reduce the M1/M2 ratio. The qPCR results (Fig. 5D, E) showed the same trends, i.e., AnSC-exos treatment positively stimulated CD206 expression and negatively stimulated iNOS expression in RAW264.7 cells compared to the rest of the groups; notably, the differences between the AnSC-exos group and the hBMSC-exos group were significant. These results demonstrate that AnSC-exos can restore the balance of the M1/M2 ratio disrupted by excessive ROS, have strong anti-inflammatory activity, and this activity is even stronger than that of hBMSC-exos.

**Fig. 6:**
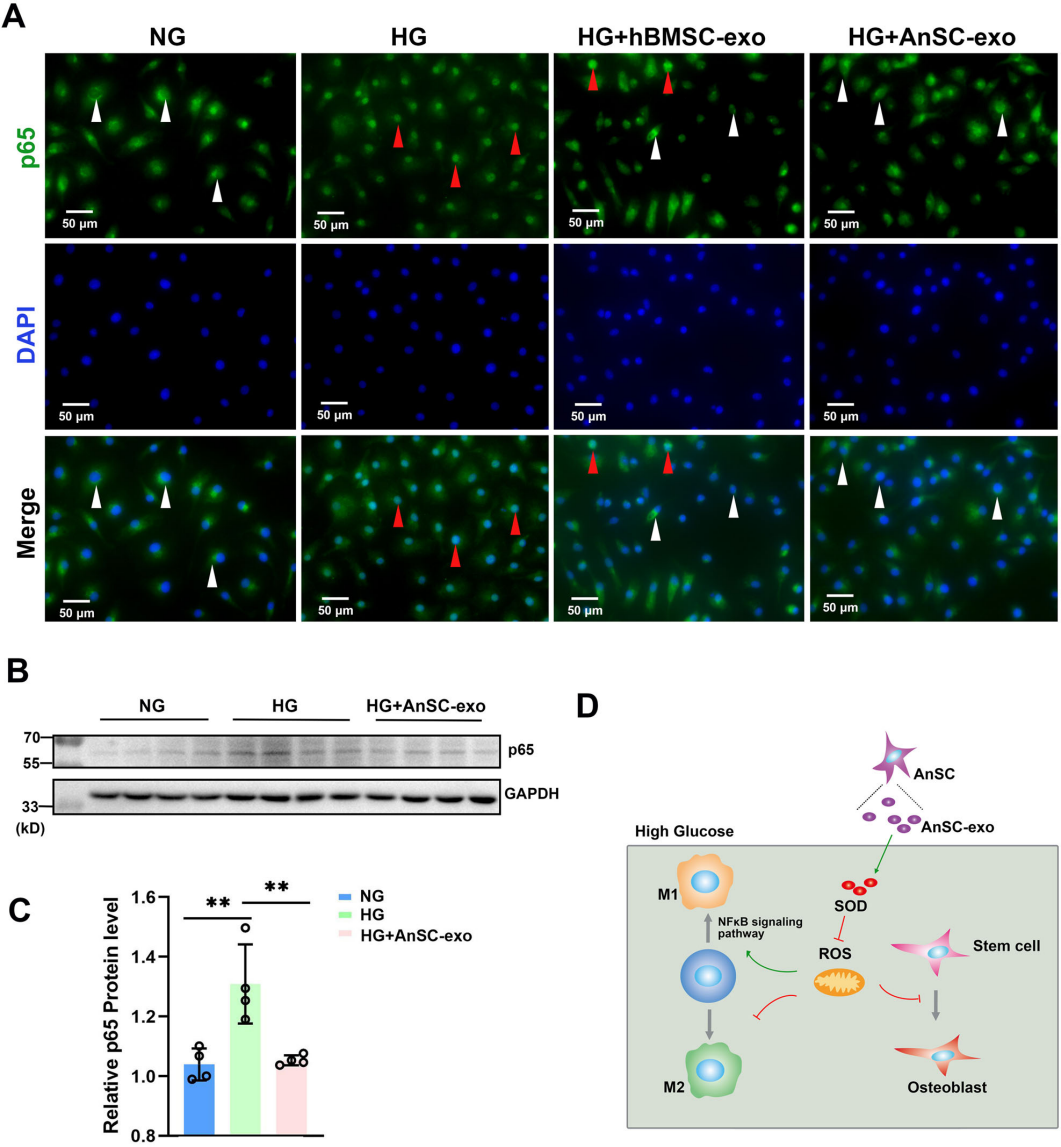
Effects of AnSC-exos on p65 translocation and Schematic illustration. **A** Representative images of p65 expression via immunofluorescent staining. Note that HG treatment induced p65 translocation from cytoplasm (white arrowheads) to nuclei (red arrowheads). **B** Western blot analysis of p65 protein expression. **C** Relative expression levels of p65 protein. Error bar represents the mean ± SD (*n* = 4). **p < 0.01. **D** Schematic illustration of the molecular mechanism underlying the effective treatment of the periodontitis in DM by AnSC-exos.

To explore the molecular mechanism underlying the anti-inflammatory activity, immunofluorescent staining and western blotting (WB) for RAW264.7 cells were performed to detect the effect of the HG environment on the expression of p65, a key factor in the NF-κB pathway [44]. As shown in Fig. 6A, HG treatment effectively induced translocation of p65 from the cytoplasm to the nucleus in the HG group, indicating the NF-κB pathway is activated; whereas a much smaller number of p65 translocations was observed in the hBMSC-exos group; notably, the process of p65 translocation was not detectable in the AnSC-exos group. The WB results showed that the expression level of p65 protein in the HG group was significantly higher than that in the NG group. However, AnSC-exos treatment reversed this trend, that is, it decreased the p65 protein level of RAW264.7 cells under the HG condition (Fig. 6B, C and Figs. S1–S2). These results indicate that both AnSC-exos and hBMSC-exos can effectively suppress the process of p65 translocation and expression upregulation induced by HG treatment and thus inactivate the NF-κB transduction pathway, and this suppressive effect of AnSC-exos is more significant than that of hBMSC-exos.

## Discussion

This study provides the first evidence that exosomes derived from antler stem cells (AnSC-exos) can effectively restore periodontal homeostasis and promote alveolar bone repair/regeneration in a rat model of diabetic periodontitis. Critically, AnSC-exos exhibited better therapeutic efficacy than human bone marrow MSC-derived exosomes (hBMSC-exos) in some key aspects, such as improving the BV/TV parameter, enhancing osteogenic differentiation, and promoting macrophage M2 polarization. Our findings reveal that the restorative effects of AnSC-exos on diabetic periodontitis are mediated through dual mechanisms: enhancing osteogenesis and mitigating inflammation via ROS scavenging.

It is reported that one way for bone regeneration in situ is the recruitment and proliferation of resident MSCs [2, 35, 45, 46]. Cell recruitment occurs during the initial stage of natural bone repair and plays a crucial role throughout the process [47]. However, the high-glucose (HG) condition in DM negatively impacts cell migration and proliferation of resident MSCs [2], and MSCs exhibit reduced osteogenic capability under diabetic conditions [48, 49]. In the present study, we found that AnSC-exos reversed the negative effects caused by the HG environment, including MSC proliferation, migration, survival, and osteogenic differentiation processes essential for periodontal bone regeneration. Notably, AnSC-exos not only restored but, in some assays (e.g., ALP activity, mineralization nodule formation), even surpassed the osteogenic potential observed under NG conditions, outperformed hBMSC-exos in some key aspects. This suggests that AnSC-exos likely deliver potent bioactive substances that can effectively stimulate osteogenesis.

There has been consensus that dysregulation of MSCs in DM is mainly attributed to the excessive production of ROS under the HG environment. In normal conditions, ROS plays a crucial role in a variety of cellular functions [50, 51]. However, excessive ROS accumulation under hyperglycemia is detrimental and a well-established inducer of oxidative stress, triggering mitochondrial dysfunction and caspase-dependent apoptotic pathways, ultimately leading to MSC death [52]. In this study, by neutralizing HG-induced ROS overload, AnSC-exos effectively alleviates oxidative damage and consequently promotes MSC survival and reduces cell death, which is fundamental for maintaining a functional MSC pool necessary for bone repair/regeneration.

Critically, the detrimental effects of HG-induced ROS extend beyond cellular damage: excessive ROS also acts as a key inflammatory mediator, activating redox-sensitive signaling pathways and fueling a chronic inflammatory state that further disrupts tissue homeostasis and impedes regeneration. In this study, AnSC-exos significantly attenuated the inflammatory milieu characteristic of diabetic periodontitis, effectively reduced intracellular ROS levels in macrophages, and enhanced superoxide dismutase (SOD) expression in the HG condition. This potent ROS-scavenging activity by SOD underpinned several anti-inflammatory effects: shifting macrophage polarization towards the reparative M2 phenotype (while suppressing the pro-inflammatory M1 phenotype), and lowering inflammatory cytokine levels both in vitro and in vivo. Furthermore, we identified the key underlying molecular mechanism: AnSC-exos significantly inhibited the HG-induced nuclear translocation of p65, the major subunit of the pro-inflammatory NF-κB transcription factor. Given that ROS acts as an activator of the NF-κB pathway [53, 54], our data strongly suggest that AnSC-exos exert their anti-inflammatory effects primarily via quenching excessive ROS, thereby preventing ROS-dependent NF-κB activation and subsequent inflammation amplification.

The superior efficacy of AnSC-exos over hBMSC-exos in some key aspects might be attributed to their specific contents, which is stemmed from their developmental origin. Cranial neural crest cells (CNCCs) are a pluripotent cell population and are subject to massive migration during embryogenesis [55]. They can form various types of tissues and cells, including tooth, bone, cartilage, nerve, and blood vessel, as well as deer antler [56]. Both AnSCs and DMCs are derived from a sub-population of CNCCs, and both exhibit certain similarities in transcriptional characteristics and differentiation potential [24]. This shared embryonic lineage suggests that AnSCs possess inherent molecular programs highly compatible with, and potentially optimized for, regenerating neural crest-derived tissues like the periodontium. This concept of “tissue-specific” MSC superiority aligns with previous reports that MSCs sharing embryonic origin with the target tissue often exhibit enhanced regenerative potential for that tissue [32, 57, 58]. For example, injured bones preferentially heal using MSCs of the same embryonic origin [16, 47]. Based on the above facts, we strongly believe that AnSCs hold greater promise in promoting periodontal repair than any other currently using MSCs. In our previous studies, we have confirmed that the paracrine factors of AnSCs effectively stimulated alveolar bone (AB) regeneration in a surgically-created AB defect model, and inhibited inflammation of the gingival tissue in an experimental model of periodontitis [16]. In the present study, we found that amongst these paracrine factors, AnSC-exos may be the key component for periodontal tissue repair, and further confirmed that AnSC-exos can significantly promote the restoration of periodontal homeostasis and inhibit periodontal bone resorption in severe periodontitis in DM. Compared with stem cells per se, exosome application rarely induces an immune response. Therefore, we believe that AnSC-exos can be developed as a potential cell-free remedy for effective treatment of diabetic periodontitis in dental clinical practice.

In conclusion, AnSC-exos represent a highly promising therapeutic candidate for the treatment of diabetic periodontitis. Via simultaneously countering the effects of two primary pathological factors: impaired osteogenesis and excessive ROS-driven inflammation, AnSC-exos significantly restored periodontal tissue homeostasis. Their overwhelming efficacy, which even surpasses conventional hBMSC-exos, highlights the potential of leveraging tissue-specific stem cells of shared embryonic origin. Compared to stem cell transplantation, exosome-based therapy offers distinct advantages, including reduced immunogenicity, lower tumorigenicity risk, easier storage and handling, and potentially better targeting capabilities. Future research should focus on optimizing AnSC-exos isolation procedure, dosage and characterization protocols; exploring targeted delivery strategies to bone lesions; and validating the long-term efficacy and safety in large animal models of diabetic periodontitis. The successful translation of AnSC-exos therapy could provide a novel and promising strategy for managing the challenge of diabetic bone disorders, such as diabetic periodontitis.

## Material and methods

### Preparation of AnSC-exos and animal experiments

AnSC-exos were obtained in our recent study [30]. The design of the animal experiments was approved by the Animal Ethics Committee of Changchun Sci-Tech University (No. CKARI202402). For the creation of diabetic rat models of periodontitis, a total of 26 4-week-old male Sprague Dawley (SD) rats (100–150 g) were used, of which 24 were randomly divided into four groups (*n* = 6 per group): non-diabetic periodontitis group (Ligation), diabetic periodontitis group (Control), diabetic periodontitis group with AnSC-exos treatment (AnSC-exos), and diabetic periodontitis group with hBMSC-exos treatment (hBMSC-exos). The remaining two rats were used as a normal control group without any treatment (Intact) and were used for micro-CT and histological analyses.

Type-2 diabetes animal models (T2DM) were constructed as previously described [2]. Briefly, rats were fed with a high-fat diet for 4 weeks. Thereafter, the rats were injected intraperitoneally with STZ dissolved in sodium citrate buffer (pH 4.5, 35 mg/kg). Two weeks after injection, the rats were fasted overnight and fasting blood sugar was measured the next morning. Rats with fasting blood glucose higher than 11.1 mmol/L showed polyuria, polydipsia and weight loss, suggesting that the establishment of the diabetic model was successful. Then, models of periodontitis were constructed using T2DM and non-T2DM rats based on previous studies [32]. Briefly, rats were anesthetized by placing them in a sealed container with a 4% (v/v) isoflurane flow. A stainless-steel wire with a diameter of 0.20 mm was fastened around the second maxillary molar. One week after ligation, PBS was injected into the periodontal pocket every other day after periodontitis was induced in both the Ligation and Control groups; AnSC-exos (50 μg/ml) and hBMSC-exos (50 μg/ml) were injected (10 μL) into the periodontal pocket every other day in the AnSC-exos and AnSC-exos groups. Specifically, PBS/exo was injected with a 500 μL syringe into the intrabuccal, distal buccal, proximal palatal, and distal palatal regions of the second molar with a total volume of 10 μL per sample.

The experimental rats were euthanized 3 weeks later. Maxillary alveolar bone was dissected and fixed in 4% paraformaldehyde for histological examination and Microcomputed Tomography (Micro-CT) analysis.

### Micro-CT analysis

Micro-CT analysis was performed as previously described [16]. Samples were collected and fixed in 4% paraformaldehyde for 48 h, and then transferred into 70% ethanol. The samples were scanned using a high-resolution Micro-CT (Scano Medical AG, Bassersdorf, Switzerland) with the acquisition protocol (70 kV, 150 mA, 10-μm increment). The scanned files were reconstructed and analyzed using the Mimics Innovation Suite software (Materialize, Belgium). The shape and size of the tissue volume used for quantitative analysis were fixed, and each sample was analyzed in the same region to minimize errors.

### Histological examination

Rat maxillae samples were decalcified in 5% methanoic acid for 1 week, then embedded in paraffin and cut at 5 μm thickness. For histology examination, the sections were deparaffinized, rehydrated, and stained with hematoxylin-eosin (H&E); subsequently, immunohistochemistry (IHC) staining was performed. In IHC, primary antibodies included rabbit anti-rat IL-1β (1:200, Proteintech, 26048-1-AP) and rabbit anti-rat IL-10 (1:200, Affinity, DF6894). Images were captured under a slice scanner (M8 microscope and scanner, PreciPoint, Germany). The integrated optical density (IOD) value and positive area occupancy (area%) were analyzed using ImageJ. Three samples were used for analysis per group.

### Cell proliferation and migration

rBMSCs were obtained in our recent study [16] and divided into four groups: (1) the NG group, the cells were cultured in normal-glucose (5.5 mM) medium; (2) the high-glucose (HG) group, the cells were cultured in HG (100 mM) medium; (3) the NG + hBMSC-exos group, the cells were cultured in HG medium and treated with hBMSC-exos (10 μg/ml); or (4) the HG + AnSC-exos group, the cells were cultured in HG medium supplemented with AnSC-exos (10 μg/ml). Cell proliferation was measured using the keyFluor594 Click-iT EDU Kit (KeyGEN BioTECH, China) according to the manufacturer’s instructions. The specific fluorescent staining was conducted under a fluorescent microscope (EVOS M5000, Thermo Fisher, USA). The number of EDU-positive stained cells was analyzed using Image-Pro Plus software, and the cell migration distance was analyzed using ImageJ software.

### Cell migration

For cell migration, rBMSCs were seeded in 24-well plates at a density of 2 × 10^4^ cells/well and incubated until confluence. These confluent cells were then scratched with a sterile pipette tip to create a stripe with 0.3–0.5 mm in width. Culture medium was then immediately removed, and cells were stained with Calcein AM (Beyotime, China) according to the manufacturer’s instructions. Images were captured using a fluorescent microscope (EVOS M5000, Thermo Fisher, USA). Then medium was replaced by (1) NG (5.5 mM) medium, or (2) HG (100 mM) medium, or (3) HG (100 mM) medium supplemented with hBMSC-exos (10 μg/ml), or (4) HG (100 mM) medium supplemented with AnSC-exos (10 μg/ml). Calcein AM staining was performed after culturing for a further 48 h, and images were captured. The width of the stripe was measured using ImageJ software, and the cell migration rate was calculated as the percentage of the original scratch width that decreased at each given time point.

### Cell viability assay

rBMSCs were seeded in 24-well plates at a density of 2 × 10^4^ cells/well and incubated in (1) NG (5.5 mM) medium, or (2) HG (100 mM) medium, or (3) HG (100 mM) medium supplemented with hBMSC-exos (10 μg/ml), or (4) HG (100 mM) medium supplemented with AnSC-exos (10 μg/ml). After 48 h of culture, the living and dead cells were identified by calcein/PI cell viability/cytotoxicity Assay Kit (Beyotime, China) according to the manufacturer’s guidelines. Then, the cells were examined under a fluorescent microscope (EVOS M5000, Thermo Fisher, USA). The number of live/dead cells was analyzed using Image-Pro Plus software.

### Osteogenic effects and alizarin red S staining

rBMSCs were cultured in osteogenic medium and divided into four groups as described in Method 4; the medium was changed every 3 days. After 21 days of culture, the rBMSCs were washed and fixed in 4% paraformaldehyde for 15 min. 0.2% Alizarin Red solution (Solarbio, Beijing, China) was added to the wells, then incubated for 30 min. After washing twice with deionized water, surface staining was photographed under a microscope. For quantitative analysis, the mineralized nodules were dissolved in 500 μl 2% cetylpyridinium chloride for 1 h, then the OD values of the solutions were measured at 560 nm using a spectrophotometer.

### Alkaline phosphatase staining

After 7 days of osteogenic induction culture, ALP staining and ALP activity were respectively performed using the Alkaline Phosphatase Stain Kits (Solarbio, China) and Alkaline phosphatase assay kit (Nanjing jiancheng Bioengineering Institute, China), according to the manufacturer’s instructions.

### Intracellular ROS measurement

For rBMSCs, cells were seeded in 48-well plates at a density of 1 × 10^4^ cells/well and divided into four groups as described in Method 4. After 24 h of culture, the ROS detection kit (Beyotime, China) was used to measure the levels of intracellular ROS. Raw264.7 cells were cultured in 48-well plates for 24 h with LPS stimulation, then ROS levels were detected using the ROS detection kit according to the manufacturer’s instructions. In brief, culture medium was removed, cells were incubated in DCFH-DA staining solution for 20 min, then washed three times with serum-free medium. The cells were observed with a fluorescent microscope (EVOS M5000, Thermo Fisher, USA), and the images were captured. ROS intensity was analyzed using ImageJ software.

### qRT-PCR

Total RNA from cells was isolated using RNA simple Total RNA Kit (TIANGEN, China) according to the manufacturer’s instructions. Total RNA was reverse-transcribed into cDNA using the cDNA Synthesis Kit (Takara, Japan). The specific primers, based on the DNA sequences located in the gene coding regions, were designed using NCBI (Table S1). Glyceraldehyde-3-phosphate dehydrogenase was used as an endogenous control. The SYBR Kit (TransGen Biotech, Beijing, China) was used in the quantitative real-time PCR (qRT-PCR) assay according to the manufacturer’s instructions. Relative expression was calculated using the 2^−ΔΔCT^ method to assess the fold change in expression levels of the target genes.

### Immunofluorescence staining

Immunofluorescence staining was performed as described in previous studies [12]. In brief, cells were fixed in 4% paraformaldehyde for 20 min, permeabilized in 0.4% Triton X-100 for 10 min, and then blocked with 3% BSA for 1 h at room temperature. Primary antibodies (rabbit anti-mouse CD206, 1:200, Affinity, DF4149; rabbit anti-mouse iNOS, 1:200, Affinity, AF0199; rabbit anti-mouse p65, 1:200, Abcam, ab32536) were added, and cells were incubated at 4 °C overnight. Cells were then washed three times and incubated with secondary antibodies at room temperature for 1 h. The cell nuclei were counterstained with DAPI. The images were captured under a fluorescent microscope camera (EVOS M5000, Thermo Fisher, USA).

### Protein preparation and western blotting

Total cellular proteins were extracted, and protein concentrations were measured using a BCA protein assay kit (Beyotime, China). For each sample, 10 and 30 µg of protein were separated by 10% sodium dodecyl sulfate-polyacrylamide gel electrophoresis (SDS-PAGE) and then transferred onto 0.2-µm PVDF membranes. The membranes were blocked in TBST containing 5% nonfat dry milk at room temperature for 2 h, followed by incubation with specific primary antibodies overnight at 4 °C: rabbit anti-p65 (1:500, Abcam, ab32536). After washing, the blots were probed with horseradish peroxidase-conjugated secondary antibodies, detected using an enhanced chemiluminescence system, and imaged.

### Statistical analysis

The results are presented as mean ± SD. Statistical significance was evaluated using GraphPad Prism 9.0.1 (GraphPad Software, La Jolla, CA) software. The comparisons of single and multiple variables were, respectively, performed using a one-way or two-way ANOVA, and Student’s *t*-test was used to compare two variables. Values were set at *p* < 0.05 for statistical significance.

### Ethics statement

All experimental procedures were conducted in accordance with the relevant ethical. guidelines and regulations approved by the Animal Ethics Committee of Changchun Sci-Tech University (Permit Number: No. CKARI202402).

## Supplementary information

Table S1

Figure S1

Figure S2

## Data Availability

All data generated or analyzed during this study are included in this published article and its supplementary information files. Additional data available from the corresponding author on reasonable request.
