# Supplementary figures and images for: Antler stem cell-derived exosomes restore periodontal homeostasis in a rat model with diabetic periodontitis through enhancing ROS scavenging and osteogenesis
Source: Cell Death Discov. 2025 Nov 3;11:500. doi: 10.1038/s41420-025-02800-6 (PMC12583464; doi:10.1038/s41420-025-02800-6)

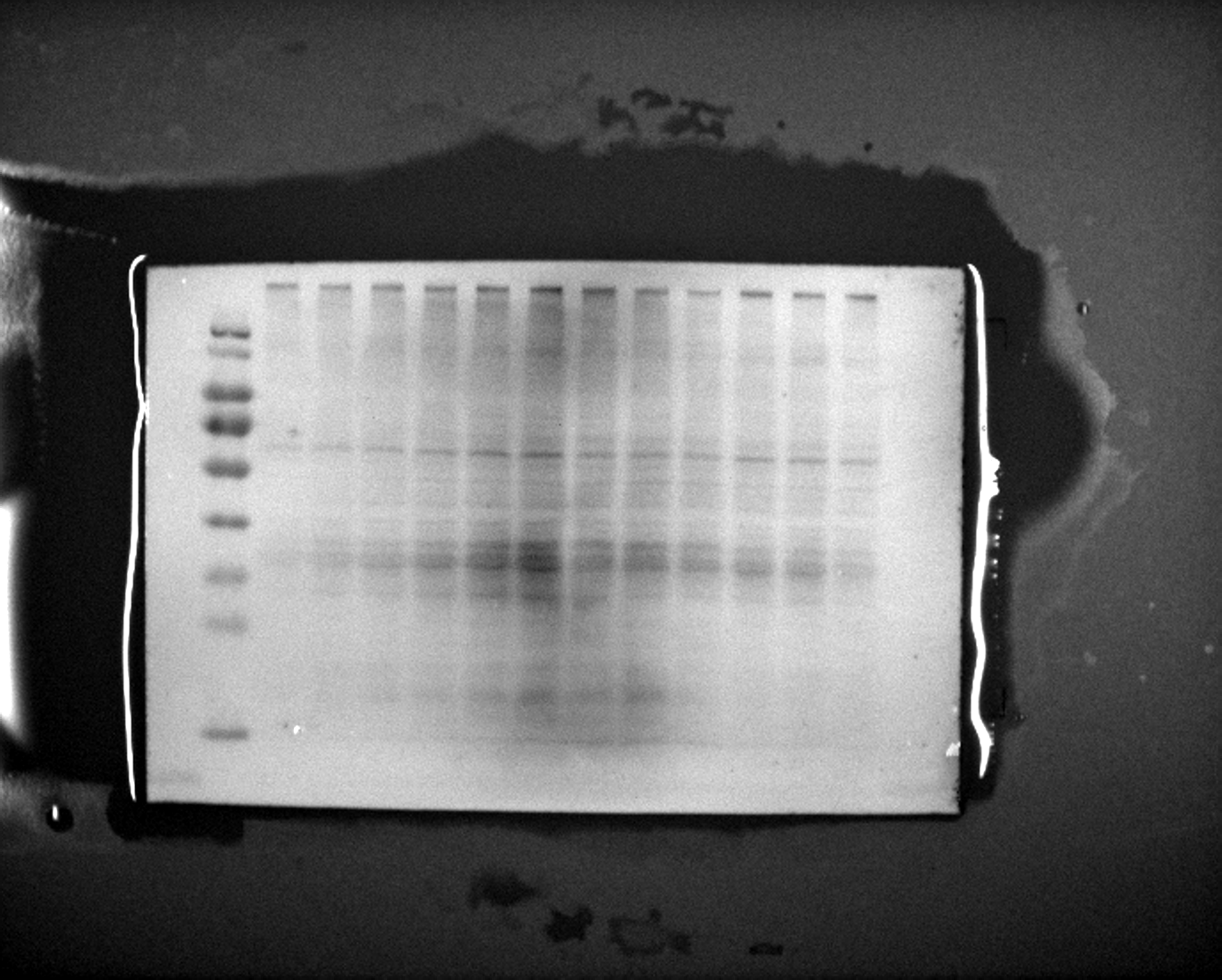

Supplement: Supplementary file 2 — Figure S1 [file 41420_2025_2800_MOESM2_ESM.tif]

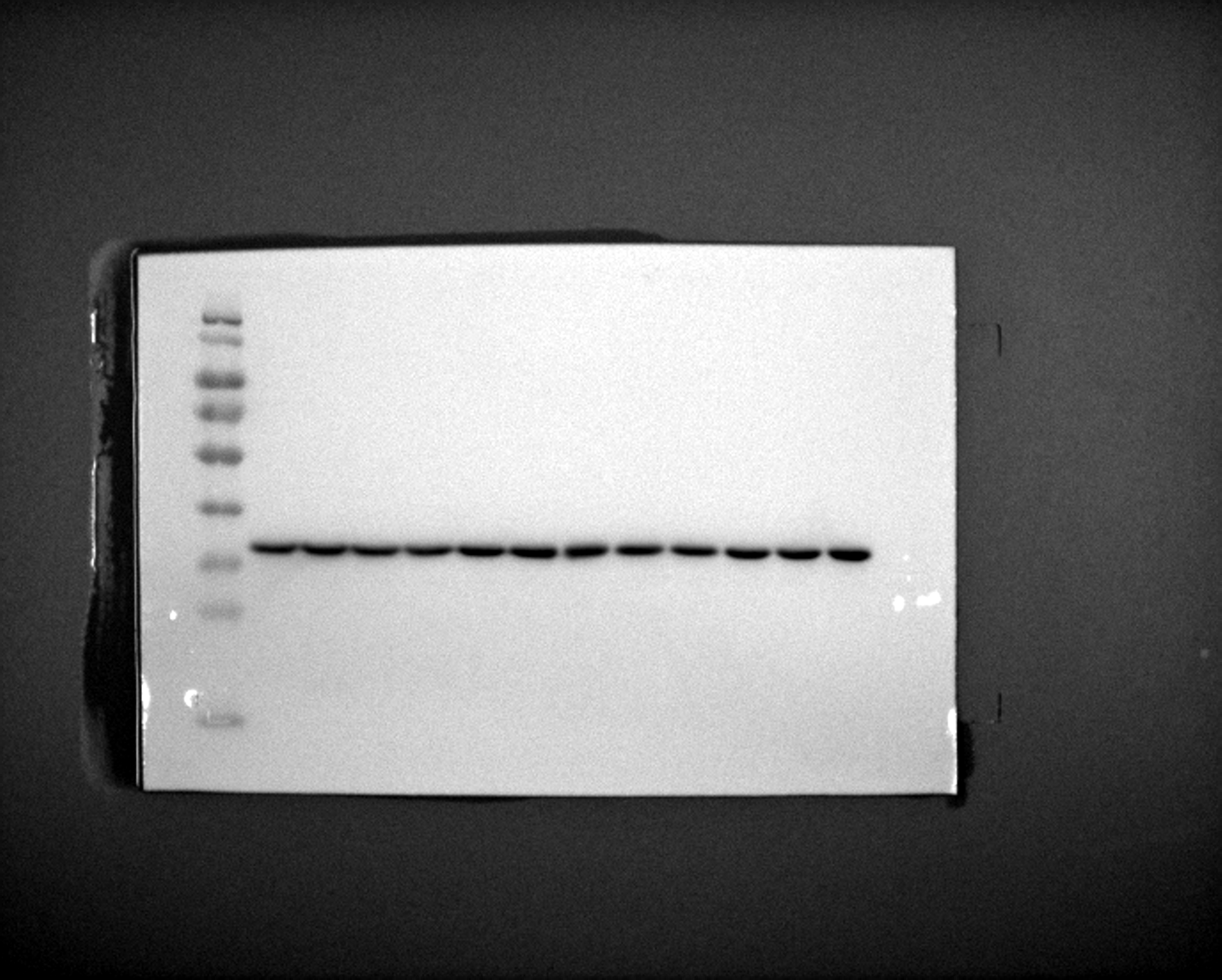

Supplement: Supplementary file 3 — Figure S2 [file 41420_2025_2800_MOESM3_ESM.tif]
